# Retrospective evaluation of oral complications following radiotherapy and chemoradiation in patients with head and neck Cancer

**DOI:** 10.1038/s41598-025-10598-7

**Published:** 2025-07-09

**Authors:** Radhika R. Pai, Ravikiran Ongole, Sourjya Banerjee

**Affiliations:** 1 Fundamentals of Nursing Department, Manipal College of Nursing, Manipal Academy of Higher Education, Manipal, India; 2 Department of Oral Medicine and Radiology, Manipal College of Dental Sciences Mangalore, Manipal Academy of Higher Education, Manipal, India; 3 Department of Radiation Oncology, Kasturba Hospital Mangalore, Manipal Academy of Higher Education, Manipal, India

**Keywords:** Postradiotherapy, Chemoradiation, Oral complications, Head and neck cancer, Cancer, Head and neck cancer, Chemotherapy, Radiotherapy

## Abstract

Patients receiving head and neck radiotherapy/chemoradiation may experience a notable and frequent decline in their oral health due to oral complications, including restricted mouth opening, oral mucositis, dry mouth, halitosis, infection, and oral pain. The main aim of this study was to describe the oral complications arising from post-Radiotherapy/chemoradiation among HNC patients through a retrospective survey of 80 patient records. This study collected patients’ comorbid illness and treatment details alongside oral complications. The record review included one-year follow-up details of HNC patients post-completion of radiotherapy/chemoradiation. Among the 80 patients who completed head and neck radiotherapy, 23 experienced interrupted treatment. Among them, most (15%) did not tolerate chemotherapy, which is part of chemoradiation. Thirty-four patients were followed up for oral complications. Most patients experienced symptoms of restricted mouth opening 12(15%), mucositis 6(7.5%), and oral pain 5(6.25%) as oral complications during the subsequent follow-up. Approximately 14 patients reported HNC recurrence. These postradiotherapy/chemoradiation-related oral complications pose a difficult challenge to patients and their caregivers. Awareness of such oral complications will enable healthcare providers to recognize and institute early interventions to provide patients with a better quality of life.

HNC typically begins in the oral cavity, pharynx, or larynx, which are all parts of the upper aerodigestive tract. The function and appearance of this area are essential to the patient’s sense of self and quality of life^1^. Oral side effects from chemotherapy for any cancer or radiotherapy to the head and neck might impair a patient’s quality of life, health, and capacity to finish prescribed cancer treatment^2^. Squamous cell carcinoma (SCC) of the head and neck is the sixth most common tumor and accounts for nearly 90% of all upper aerodigestive tract cancers. Damage to and fibrosis of the masticatory muscles appear to be connected to the loss of function and range of motion in the mandible following radiotherapy^3^. Patient characteristics, the leading site, the clinical stage, and tumor resectability influence the radiotherapy/chemoradiation method plan^4^. Tissue fibrosis, salivary gland dysfunction, heightened susceptibility to mucosal infections, neuropathic pain, sensory problems, dental caries, and periodontal disease are among the long-term, chronic effects of head and neck radiotherapy^1,5^.

Radiotherapy and Chemotherapy are associated with various complications, affecting the mouth, hard and soft tissues, and associated structures such as the salivary glands^6,7^. These complications, from initial oral mucositis to xerostomia to oral mucositis, substantially influence patients’ quality of life and management plans^8^. Most patients receiving radiotherapy for cancer of the head and neck experience oral mucositis (OM), an acute response to treatment^5^. The symptoms of abrupt severe OM include significant pain and difficulty swallowing and eating. Oral mucositis is linked to increased pain, a falling-per-performance status, an inability to eat, and the need for feeding tubes, all of which decrease quality of life^9^. In addition to treatment, patient characteristics such as advanced age, sex, smoking status, alcohol consumption, altered oral intake, preexisting periodontal disease, low body mass index, poor functional status, low leukocyte count, advanced disease and stage, a history of severe mucositis in the past, and various comorbid conditions have also been linked to the incidence of OM. Hospitalization, feeding tube placement, and treatment breaks are also linked to OM incidence^10^.

One of the most frequent side effects of radiotherapy for cancer of the head and neck is xerostomia, or dry mouth, a decrease in salivary function as the radiation fields, including the salivary glands, can suffer permanent damage^11^. In addition to drastically reducing the quality of life for cancer patients who may yet receive a cure, xerostomia can cause serious, chronic dental problems. Xerostomia impairs oral cavity function, making it harder to chew and swallow food and impairing speech. Furthermore, low salivation increases the risk of radiation caries and infections such as candidiasis and gingivitis^12,13^.

Most patients experience partial or total taste loss after radiotherapy^14,15^. When patients receive radiotherapy alone, the incidence of taste abnormalities is 66.5%; however, when they also receive chemotherapy, the frequency increases to 76%^16^. HNC and its treatment are thought to impact food preferences and eating habits^17^. The inability to consume food can lead to several complications when persistent severe dysgeusia persists. These include vitamin deficiencies, food aversions, weight loss, malnutrition, increased use of sweeteners and complementary spices, ineffective treatment responses, and adverse effects on quality of life^18,19^.

Dental caries can occur in patients receiving cancer therapy, primarily due to xerostomia. Patients who have more posteriorly positioned oral tumors, such as those near the base of the tongue, are at risk because it is impossible to prevent radiation to one of the parotid glands^5^. Months or years after radiotherapy ends, delayed side effects, including trismus (from muscle fibrosis), loss of salivary gland function, widespread dentition degradation from radiation caries, and osteoradionecrosis, appear^20^. The impact of ionizing radiation on the TMJ has significant clinical implications, resulting in diminished oral functions, osteoradionecrosis, and degenerative TMJ disorders^21^. This alters the composition of saliva, which causes it to lose its lubrication qualities and become dry and sensitive (xerostomia)^22^. Patients with cancer who experience dry mouth may experience problems with eating, speaking, and swallowing, as well as an increased risk of dental caries and oral candidiasis. Additionally, a patient’s nutritional and psychological well-being may be negatively impacted^23^.

This retrospective study assessed both short-term and long-term oral complications associated with radiotherapy and chemoradiation in HNC patients. We believe that this study will provide insight to health care providers so that appropriate treatment strategies to manage complications of radiotherapy and chemoradiation can be planned.

## Methodology

A retrospective survey of medical records was carried out in 2021 among 80 HNC patients who completed radiotherapy/chemoradiation between 2016 and 2020 at a tertiary care center in southern India. Following the completion of radiotherapy or chemoradiation, the oral health status of patients was evaluated over a one-year period based on their medical records. This retrospective study aimed to elucidate the oral complications and their subsequent management post-radiotherapy or chemoradiation. The study encompassed various aspects such as oral complications, referrals for palliative radiotherapy or chemotherapy, reasons for treatment interruptions, mortality rates, cancer recurrence, and the frequency of follow-up outpatient department (OPD) visits. Patients experiencing oral complications were assessed by a radiation oncologist, who provided treatment or referred them to other specialized departments based on the presenting symptoms.

The Institutional Ethics Committee of Kasturba Medical College, Mangalore (IEC KMC MLR 03–18/65), granted approval for this retrospective study. All methods were performed according to the relevant guidelines and regulations. Informed consent to participate was waived by the Institutional Ethics Committee (IEC) at Kasturba Medical College, Mangalore. The study was registered at CTRI (CTRI/2015/04/005709).

## Inclusion and exclusion criteria

Medical records from the radiation oncology department were evaluated between 2016 and 2020. All patients who received radiation/chemoradiation for HNC were included. Other cancers and those where surgery alone was performed were excluded. Additionally, the analysis did not include patients who did not opt for treatment or discontinued treatment.

The records of the patients who were included in the study were evaluated by a single researcher. The patients’ demographic details, the nature of the cancer, the treatment provided, and the complications reported both during and within the first-year follow-up periods were documented. The data collected from the patient records were analyzed via descriptive statistics.

## Results

Twenty-eight out of 80 HNC patients visited the hospital for follow-up, with an adherence to follow-up rate of 42.5%. A retrospective analysis of the records was carried out to determine the oral health status and treatment details of the patients after the completion of radiotherapy/chemoradiation.

**Table 1:** Baseline sociodemographic characteristics of patients *N* = 80.

Variables	f	%
**Age in years**		
18–55	37	46.25
56 and >	43	53.75
**Gender**		
Male	72	90
female	8	10
**Education**		
Illiterate	28	35
Primary education	31	38.75
Higher Secondary	18	22.5
Graduation	3	3.75
**Type and location of the cancer**		
Tongue	30	37.5
Buccal mucosa	10	12.5
Larynx	2	2.5
Supraglottis	7	8.75
Hypopharynx	11	13.75
Oropharynx	13	16.25
Tonsil	1	1.25
Maxilla	1	1.25
Pyriform fossa	3	3.75
Glottis	1	1.25
Nasopharynx	1	1.25
**BMI**		
Malnourished	16	20
Underweight	25	31.25
Normal	35	43.75
Overweight	4	5

In the present study, the majority, i.e., 43 (53.75%), were 56 years or older. Most patients (72, 90%) were males. Most patients, 31 (38.75%), reported having a primary education. Among the patients, 30 (37.5%) had tongue cancer. Most patients had an average BMI of 35 (43.75%) before radiotherapy. (Table 1)

**Table 2:** Description of the treatment and TNM classification and staging details *N* = 80.

Variables	f	%
**Type of treatment**		*N* = 80
Radiotherapy	30	37.5
Chemoradiation	50	62.5
**Radiotherapy: Dose in Grays (Gy)**		*N* = 80
60	7	8.75
66	10	12.5
70	63	78.75
**Chemoradiation Agent**		*N* = 50
Cytoplatin	19	38
Cisplatin	2	4
Kemocarb	18	36
Gefitinib	4	8
Chemoplat	6	12
Unicarb	1	2
**TNM classification**		*N* = 80
**Tumor**		
T1	3	3.75
T2	16	20
T3	26	32.5
T4	35	43.75
**Node**		
N0	24	30
N1	4	5
N2	46	57.5
N3	6	7.5
**Metastasis**		
Mx	66	82.5
M0	14	17.5
**Stage of cancer**		
I Early localized	2	2.5
II Early locally advanced	16	20
III Late locally advanced	26	32.5
IV Metastasized	36	45

Overall, 50 (62.5%) HNC patients received chemoradiotherapy. Most of the 50 patients who received chemoradiotherapy were treated with cytoplatin (19; 23.75%) and kemocarb (18; 22.5%) as chemotherapy agents. With respect to the TNM classification, 35 (43.75%) patients had HNC, 46 (57.5%) had N2 cancer, and 66 (82.5%) had Mx cancer. Most of the patients, 36 (45%), had stage IV cancer during diagnosis. Among the 44 patients with comorbidities, 63 (78.75%) had diabetes. (Table 2)

**Table 3:** Follow-up details of HNC patients one year after the completion of radiotherapy.

Follow-up details	f	%
**Palliative treatment (n= 10)**	*n* = 10	
Palliative radiotherapy	2	2.5
Palliative chemotherapy	8	10
**Reasons treatment interruptions (n= 23)**	*n* = 23	
Chemo not tolerated	12	15
Discharge against medical advice (DAMA)	5	6.25
Alcohol withdrawal symptoms	1	1.25
Pancytopenia	2	2.5
Hyperbilirubinemia	1	1.25
Fall injury	1	1.25
Neck wound	1	1.25
**Causes of death (n = 6)**	*n* = 6	
Sepsis	1	1.25
Aspiration pneumonia	4	5
Cardiac arrest	1	1.25
**Recurrence of cancer (n= 14)**	*n* = 14	
Local	7	25
Regional	3	10.71
Locoregional	2	7.14
Distant	2	7.14
**Number of follow up OPD visits(n=22)**	*n* = 24	
Once	2	2.5
Twice	8	10
Thrice	3	3.75
Four times	6	7.5
Five times	2	2.5
Six times	1	1.25
**Oral complications elicited during follow-up**	Number of symptoms = 34	
Pain	5	6.25
Swallowing difficulty	2	2.5
Restricted mouth opening	12	15
Mucositis	6	7.5
Fungating ulcer	1	1.25
Dysphagia	2	2.5
Odynophagia	2	2.5
Halitosis	1	1.25
Tongue protrusion restricted	1	1.25
Infection	2	2.5

Among 80 patients who completed radiotherapy/chemoradiation, 28 patients out of 80 visited the hospital for follow-up, with a follow-up rate of 35%. The percentages in this table are relative to *N* = 80, and oral complications elicited during the follow-up are relative to the number of symptoms.

Among all patients who came to the hospital OPD for follow-up, a total of 10 patients were referred for palliative care, 2 for palliative radiation, and 8 for palliative chemotherapy. Approximately 23 patients had interrupted treatment for various reasons, such as DAMA, alcohol withdrawal symptoms, pancytopenia, hyperbilirubinemia, fall injury, and neck wounds, and many of them (12, 15%) did not tolerate chemotherapy. Among the six patients who died, four died from aspiration pneumonia, and one each from sepsis and cardiac arrest. Approximately seven patients were diagnosed with local recurrence of HNC, three with regional recurrence, two with locoregional recurrence, and two with distant recurrence of HNC. Most of these problems were identified during follow-up, and the patients were referred to the ENT OPD and advised to undergo surgery or palliative Chemotherapy/Radiotherapy. On average, after the patients completed the prescribed radiotherapy/chemoradiation, they were followed up at the OPDs. Most patients experienced symptoms of restricted mouth opening 12(15%) and mucositis due to oral complications during the subsequent follow-up. (Table 3)

## Discussion

A retrospective study was conducted to describe the oral health status of patients before, during, and after radiotherapy/chemoradiation for treating HNC. Among 80 patients, only 34 came for the follow up, resulting in a decreased follow-up rate. A similar reduction in follow-up was reported in a study among 131 patients who had completed radiotherapy; 109 patients were followed up for at least 3 months after the end of RT, with a mean follow-up time of 120 days.

This retrospective survey of patient records reported complications such as pain, swallowing difficulty, restricted mouth opening, mucositis, fungating ulcers, dysphagia, oddsophagia, halitosis, restricted tongue protrusion, and infection during the follow-up period. Xerostomia, candidiasis, mucositis, radiation caries, and osteoradionecrosis were reported by another study as symptoms reported during follow-up^24^. The late side effects of head and neck radiotherapy include permanent loss of saliva, osteoradionecrosis, dental caries, oral cavity necrosis, fibrosis, impaired wound healing, and skin changes^7^. Hyposalivation/xerostomia, dysphagia, radiation caries, trismus, and osteoradionecrosis are the most common late adverse effects^25^.

A study carried out by Lalla et al. in 2017 reported decreased salivary flow from 1.09 to 0.47 ml/min (*p* <.0001), reduced mouth opening from 45.58 to 42.53 mm (*p* <.0001), and 8.1% experienced oral mucositis, with 3.8% developing mouth ulcers following six months of radiotherapy. At six months, there were changes in oral health-related QOL issues concerning dry mouth, sticky saliva, swallowing solid food, and taste perception (*p* ≤.0001)^26^. Among 194 patients with HNC, rapid recurrence occurred in 39 patients^27^. Most of the patients in this retrospective survey were followed up for complaints of restricted mouths. In the literature, restricted mouth opening or trismus is often encountered in patients with HNC who undergo radiotherapy, especially when a patient has a considerable tumor close to their masticatory muscles and needs long-term cancer therapy^28,29^.

## Limitations

This study has several limitations. First, its retrospective nature limits the ability to control for confounding variables or ensure consistent data collection. Second, the low follow-up rate (42.5%) may bias results toward patients with severe symptoms, potentially underestimating complication prevalence. Third, the absence of standardized grading for oral complications (e.g., CTCAE for mucositis) limits comparability with other studies. Finally, the lack of a control group prevents conclusions about causality between radiotherapy/chemoradiation therapy and observed complications.

## Conclusion

In conclusion, most of the patients who underwent radiotherapy were lost to follow-up, indicating significant unattended and unreported health concerns in HNC patients. In this cohort, restricted mouth opening and chemotherapy intolerance were observed in a subset of patients, highlighting potential challenges in treatment adherence and post-radiotherapy oral health. Further prospective studies are needed to confirm these findings and evaluate their impact on long-term quality of life. Patients and their caregivers face challenging obstacles because of several side effects. Early assessment and addressing these adverse effects can significantly increase patients’ well-being, longevity, and standard of living. The results concerning postradiation oral health concerns in this study highlight how important it is to monitor how radiotherapy affects healthy oral tissues, how preexisting conditions affect oral health, and how they progress. Many patients do not return for follow-up, which is a significant concern, as it indicates an unwillingness to return to complete the treatment.

Interruptions in the treatment plan signify the need for timely identification and management of side effects to ensure the patient’s quality of life following treatment for HNC. “A comprehensive follow-up strategy is imperative to mitigate complications arising from radiotherapy (RT). Routine trismus screening post-RT is essential for early detection of complications, including mucositis and oral pain, thereby preventing their progression during cancer treatment. Healthcare providers for HNC patients must adhere to a carefully thought-out follow-up system for timely assessment and addressing of oral complications.

## Data Availability

The datasets used and/or analyzed during the current study are available from the corresponding author on reasonable request.
